# Telework and Face-to-Face Work during COVID-19 Confinement: The Predictive Factors of Work-Related Stress from a Holistic Point of View

**DOI:** 10.3390/ijerph19073837

**Published:** 2022-03-23

**Authors:** Iduzki Soubelet-Fagoaga, Maitane Arnoso-Martinez, Edurne Elgorriaga-Astondoa, Edurne Martínez-Moreno

**Affiliations:** Department of Social Psychology, Faculty of Psychology, University of the Basque Country, 20018 Donostia-San Sebastián, Spain; maitane.arnoso@ehu.eus (M.A.-M.); edurne.elgorriaga@ehu.eus (E.E.-A.); edurne.martinez@ehu.eus (E.M.-M.)

**Keywords:** confinement, work-related stress, teleworking, face-to-face

## Abstract

This article explores the socio-labor conditions in which people worked during confinement, analyzing the predictors of work-related stress, according to work modality (face-to-face or teleworking), from a holistic and quantitative (*n* = 328) point of view. To identify predictors of stress, correlational analyses and multiple hierarchical regressions were conducted with individual, organizational, and societal variables. Furthermore, to analyze the possible modulating role of gender, caregiving, and the level of responsibility in organizations in the relationship between predictor variables and work stress, the macro process of Hayes was used. Our results show that work–family conflict and ruminative thoughts predict stress in both modalities. In teleworking modality, the hours dedicated to work predicted stress, and in face-to-face modality, safety measures and perceived economic threat (tendentially). Being in charge of persons moderated the relationship between ruminative thoughts and economic threat, and stress in face-to-face. Results are discussed by identifying good practices that can improve workplace risk prevention strategies.

## 1. Introduction

During the confinement period, the work activity of millions of people was precipitously transformed [1]. Whereas some activities had to be canceled due to the restrictions imposed, others moved their jobs to their homes, with face-to-face activity only continued in cases where it was deemed essential. Specifically, in Spain, 66% of the people who continued their professional activity during confinement continued to go to their workplace, whereas 34% teleworked [2]. This represented a significant increase and qualitative change in this work modality because before the pandemic, only 4.8% teleworked [3], and it seems that teleworking was related to the professional group or the status of the job. Thus, people with positions of greater responsibility were more likely to adopt this work modality (60.6%), followed by 21.4% of people with intermediate positions, and only 18% of key workers [4]. Similarly, although telework increased during confinement, data show that people with higher occupational status had greater access to this type of work [5].

Research in the context of COVID-19 has generated many studies focused on the socio-labor conditions experienced during confinement. Most of these studies have analyzed factors that influenced the occurrence of stress or psychological distress of workers [6,7,8,9]. Whereas some have focused on telework [6,7,8,10,11], others have focused on face-to-face work, particularly on the socio-labor situation of social-health personnel [9,12,13,14]. However, fewer studies have explored both work modalities together (to our knowledge, three: [15,16,17]). Furthermore, none of these have analyzed factors related to the occurrence of stress from a holistic standpoint. This perspective allows understanding the extent of the concept of stress in its entirety and complexity [18]. In this regard, this study explores, from an integrated point of view, the explanatory factors of work-related stress and its moderating variables for both face-to-face and teleworkers. From an applied perspective, we have tried to extract lessons that, in the future, could help organizations to prevent occupational risks, and protect the health of their employees.

## 2. Conceptualization of Stress and Its Predictors

Stress has been defined as “a particular relationship between the individual and the environment that is evaluated by the individual as threatening or overwhelming his or her resources and endangering his or her well-being” [19] (p. 43). In a work context, stress has repercussions at individual and organizational levels [20], issues that, during the pandemic—and in a context oriented towards maintaining economic activity above all things —were paid less attention than in times of normality. In particular, people who continued to work during confinement (both teleworkers and those who worked outside the healthcare setting), showed high levels of distress (on a scale of 4 ≥ 3), but also reported feeling more stressed than in the context of normality [16]. Regarding the influence of work modality, the results found to date are inconclusive. Hamouche [21] found that teleworking was a cause of stress during confinement; Rodríguez et al. (2020) [17] found lower stress levels among people who combined teleworking with face-to-face work, and found no significant differences between the stress level of teleworkers and those who worked face-to-face. On the other hand, Escudero-Castillo and collaborators [15] showed that after confinement, the perceived well-being of teleworkers was lower than that of people who worked face-to-face.

To understand the causes of stress, this study started from a holistic conceptualization proposed by Durán [22], where stress in the workplace arises from the confluence of extra-organizational, organizational, and individual factors.

Extra-organizational factors include the effects of economic climate, processes of precariousness, and job instability [23]. In particular, in the context of COVID-19, the occurrence of stress has been related to the economic uncertainty resulting from the pandemic and confinement [24,25]. In this regard, Rodríguez et al. [17] showed that stress was higher among unemployed people than those who continued with their work. 

In addition, it should be considered that job insecurity and the economic threat are compounded by variables such as care responsibilities, gender, and the position that employees have in the organization. It has been found that during confinement, the economic threat was a greater concern for families with dependent minors [26]. Concerning gender, although there are relatively few gender differences in unemployment and the perceived threat of unemployment [27], women continue to be exposed to more precarious working conditions [27,28]. This situation of greater vulnerability leads us to expect that women will suffer more from the consequences of the crisis caused by the pandemic [27]. Outside the pandemic context, and in reference to the influence of occupational position, Sora et al. [23] showed that the perception of fear of dismissal is higher among people with lower-ranking positions. In the context of the pandemic, the stress caused by the economic threat was higher among people with low and middle incomes [24]. 

This extra-organizational dimension also includes work–family conflict [29,30]. Netemeyer and collaborators [29] defined this conflict as that which arises when work obligations have repercussions for family care. The pressures arising from employment through different sources generate difficulties in meeting family responsibilities, causing stress among workers [30]. In the context of confinement, with the closure of schools and without additional resources for the care of dependents, reconciliation took on significant relevance among those who continued to carry out their professional duties [31]. Most families had increased demands for child and adolescent care, and due to the existing imbalance in care responsibilities, this burden fell mainly on women [32]. 

Regarding the influence of work modality, the literature on the relationship between telework, work–family conflict, and stress is not entirely conclusive, and it appears that telework is both the cause and the solution for reducing the tension between work and care domains [33]. On the one hand, telework allows for better time management of work, family care, and leisure [34], since flexibility in organizing the working day is positively valued [35,36]. Some studies have pointed out that people who telework regularly suffer from low work–family conflict [37,38]. Other studies, however, have underlined the difficulties created by the permeability between the work–family domain [34,38]. Concerning the confinement, although some studies (e.g., [11,39]) have shown the benefits of working from home reconciling work with family life, most of them have highlighted the difficulties generated by this. In terms of benefits, Toscano and Zappalà’s [40] study showed that living with children during confinement positively moderated the relationship between overall performance and remote work productivity. Having to respond to children’s needs would probably make people more likely to work in a more motivating way. Besides, the study carried out by Xiao and collaborators [39] pointed out both the benefits and risks of working from home with children for mental health. In particular, people who worked from home and who had children in their care were shown to have greater mental well-being compared to people who had not. However, working with children from home also appeared to be a predictor of new mental health issues. Finally, Barriga Medina and collaborators [41], and Tavares and collaborators [11], showed that teleworkers suffered high work–family conflict, and that was related to the teleworkers’ burnout [41].

In addition, to explain this conflict, the number of children [37] and the gender of the teleworker must be considered [42,43]. In general, women with caregiving responsibilities tend to find it more difficult to telework [42,43], a situation that has also been noted during confinement [31]. In addition, due to the closure of schools and the increasing demand of schooling at home, and the gender imbalance in household chores, Xiao and collaborators’ [39] study showed that women teleworkers have experienced a higher risk of depression during the confinement. 

Regarding organizational factors, the predictive role of variables such as insufficient resources to cope with the task [44], lack of security in the work environment [45], or work overload [20] has been studied. Undoubtedly, the arrival of the pandemic brought about a substantial and immediate change in the way work activity continued. This is reflected in the data mentioned above concerning the growth of teleworking in a context in which workers have received little training for such work [2]. Considering this growth, it appears that the resources provided by organizations, and the capacity of employees to adapt, have been limited.

In particular, recent data show that 39.1% of teleworking people indicated that teleworking required more effort than face-to-face work [46]. Morikawa [47] states that, in the situation of confinement, the implementation of telework has occurred in a forced manner, without the workers having the necessary resources to carry out this work. In this sense, the research conducted during confinement by Tavares and collaborators [11] points out that, although teleworkers seem to have adapted easily to the sudden implementation of this modality, among the difficulties experienced is a lack of resources in terms of the necessary infrastructure for teleworking. Similarly, Ruiz-Frutos and collaborators [16] point out that the workload of most teleworkers increased during confinement, and that this influenced the emergence of stress. Nevertheless, Donati and collaborators [48] revealed that not all workers experienced teleworking in the same way. In particular, people were more comfortable working from home, and reported higher levels of well-being when they had a large experience in teleworking, and worked in large organizations. It is likely that these workers worked with greater technical and teamwork support, among others.

In the case of people whose professional activities had to be carried out from their workplace, given the situation of uncertainty caused by COVID-19, workers and unions demanded minimum safety conditions in the workplace. Despite this, 42.3% of workers indicated not working safely [16]. Safety at work is a predictor of lower stress, anxiety, and depression [49]. In the same vein, the literature review conducted by Hamouche [21] on studies published during the beginning of the pandemic (from December 2019 to March 2020) shows the importance of safety measures to ensure the welfare of face-to-face workers, noting that the lower the safety measures, the greater the stress levels.

Finally, regarding individual factors, and focusing on the pandemic situation, fear of the disease and the possibility of contagion have provoked ruminative thoughts and compulsive checking behaviors [25], causing high levels of stress [24]. Concerning gender, higher levels of risk perception [50,51] and worse self-perceived well-being [15] have been detected among women than men. In addition, given the concern to transmit the virus to the family, there was greater concern stemming from fear of contagion among those with minor dependents [26,51]. Moreover, considering the exposure to the disease, the perceived threat of health deterioration was particularly significant among those who attended the workplace [52].

## 3. Study Objectives and Hypothesis 

### 3.1. Objectives 

The objective of this study was to explore the socio-labor conditions in which people worked during confinement, analyzing the predictors of work-related stress, according to work modality (face-to-face or teleworking), from a holistic point of view. In particular, to explore the predictors of stress, we analyzed the influence of extra-organizational (perceived economic threat and work–family conflict), organizational (organizational resources available to deal with the changes at work, and the increase (or not) in workload), and individual (ruminative thoughts about the pandemic) factors.

In addition, we also sought to explore the modulating role of gender, care, the level of responsibility in organizations, and the income level in the relationships between predictor variables and work stress. In particular, we sought to explore the modulating role of gender and care responsibilities in the relationship between three predictor variables (economic threat, work–family conflict, and rumination) and work stress. As well, we analyzed the modulating role of the level of responsibility in organizations and income level in the relationships between perceived economic threat and work stress. Finally, we examined the modulating role of the organization’s size in the relationship of resources provided by the organizations for teleworking and work stress. We sought to analyze the modulating role of the variables mentioned (and not of all the variables analyzed in this study), since these are the variables that have a theoretical justification for such an analysis. 

With this, and from an applied perspective, and considering the influence of extra-organizational, organizational, and individual factors in the appearance of work-stress, the aim was to extract some lessons that could help organizations facing possible similar situations in the future. In particular, we wanted to explore how our findings could inform the development of protocols that optimize the organizational response to ensure the occupational health of their employees and prevent, in a targeted manner (depending on the type of work), the occurrence of stress among employees. 

### 3.2. Hypothesis 

Regarding extra-organizational variables, we expected that perceived economic threat would predict stress in both teleworkers and face-to-face workers (hypothesis 1) [24,25]. Furthermore, considering that fear of the economic crisis caused by the pandemic was higher among people with minor dependents [26], and that the economic consequences are greater for women [27], we predicted that taking care of a dependent person (hypothesis 2-a) and gender (hypothesis 2-b) would moderate the relationship between perceived economic threat and stress during confinement. Besides, it was expected that the level of responsibility in organizations (hypothesis 2-c) and income level (hypothesis 2-d) would moderate the relationship between stress and perceived economic threat [24]. Moreover, given that teleworkers are those with the highest professional status [4,5], we expected that perceived economic threat would be more predictive of stress in people who worked face-to-face compared with those who teleworked (3).

Work–family conflict was also expected to predict stress in both working modalities (hypothesis 4) [31,39,41]. Further, given the gender inequalities in care responsibilities and household chores, and the increased demand for child and adolescent care that confinement entailed [31,32], it was hypothesized that both gender (hypothesis 5-a) and caregiving (hypothesis 5-b) would moderate the relationship between work–family conflict and stress.

Concerning organizational variables, we anticipated that for face-to-face workers, security measures for face-to-face work would predict stress (hypothesis 6) [49]. Furthermore, given the hasty manner in which telework was established [47], and the fact that this involved greater effort on the part of teleworkers [16,46], it was expected that organizational resources allocated to telework and hours worked (hypothesis 7) would predict stress. At this level, it was expected that the organization’s size would moderate the relationship between organizational resources provided to teleworking and job stress (hypothesis 8) [48].

Concerning the individual rumination variable, it was expected to predict stress in both work modalities (hypothesis 9) [24]. Finally, given that fear of contagion was higher among women and caregivers [26,51], it was hypothesized that gender (hypothesis 10-a) and caregiver status (hypothesis 10-b) would moderate the relationship between ruminative thoughts and stress. 

## 4. Material and Methods 

### 4.1. Participants

The sample consisted of 328 people (M_e_an = 43.48 years; SD = 10.07), of whom 53.35% were teleworking, and 46.65% were working face-to-face. Of the total sample, 54.6% were women, and 20.6% of workers had caregiving responsibilities, with no differences between men and women. The level of education was high, being statistically higher in those who teleworked (master’s level) compared to those who attended the workplace (degree level) (M = 7.23; SD = 0.9 versus M = 6.36; SD = 1.08) (t = 6.85; *p* < 0.010). Among people who teleworked, the presence of women was higher (65% vs. 35%) (χ^2^ 4.51; *p* < 0.050), whereas among people who worked face-to-face, there were no gender differences (51.5% vs. 48.5%). Regarding responsibility in the organization, the level was significantly higher among people who teleworked (M = 4.04; SD = 1.59 vs. M = 3.44; SD = 1.75) than those who attended the workplace (t = 3.21; *p* < 0.010), and there were no gender differences in this regard (female teleworkers M = 4.14; SD = 1.41; male teleworkers M = 3.85 SD = 1.82 (t = 1; *p* = 0.317), and female face-to-face workers M = 3.28; SD = 1.8; male face-to-face workers M = 3.38; SD = 1.85 (t = 0.269; *p* = 0.788)). In addition, we asked about the income levels of the participants, but due to the sensitivity of the question, we were unable to collect information on this variable.

### 4.2. Procedure

After obtaining approval from the ethics committee of the University of the Basque Country (M10/2020/088), the sample of participants was recruited through an online questionnaire (Survey-Monkey platform) (Survey-Monkey, San Mateo, CA, USA) posted on social networks (Facebook, WhatsApp) (Meta Platforms, Inc, Menlo Park, CA, USA). Data collection was carried out between April and May 2020, during the confinement and state of alarm declared by the Spanish Government. Participants were informed of the study’s objective, asked for permission for the use of the data, and assured of their anonymity and confidentiality.

### 4.3. Instruments

People who teleworked and those who worked face-to-face work answered the same questions, except those related to organizational resources, where specific questions adapted to each work modality were formulated.

Sociodemographic characteristics. The following variables were included: gender (1 = “Male”, 2 = “Female”), age, taking care of a dependent person (1 = “Yes”, 0 = “No”), educational level (1 = “Basic studies not completed”; 8 = “Doctorate”), the level of responsibility in organizations based on a Likert scale (1 = “No responsibility”, 2 = Low level of responsibility, 3 = Medium level, 4 = High level, 5 = “Very high level of responsibility”), family income level (1 = less than 1000 euros per month and 5 = more than 5000 euros), and organization size measured by the number of employees (1 = 1–10 employees, 2 = 11–50, 3 = 51–100, 4 = 101–250, 5 = more than 250).

Modality of work. This was determined through one question (“What is your current work situation? “) with the following response options: 1 = “Teleworking,” 2 = “Attending my workplace”.

Job stress. This was evaluated through six items of the Stress in General Scale [53] (“To what extent do you feel the following way in relation to your job?”: “Under pressure”, “Nervous”, “Burdened”, “Calm”, “Comfortable”, “Having difficulty in fulfilling my duties”) (1 = “Not at all”, 7 = “Completely”) (α = 0. 88).

### 4.4. Extra-Organizational Variables

Perceived economic threat. This was measured by an ad hoc scale with two items (“Indicate your degree of agreement or disagreement with the following statements”: “I am afraid of the economic crisis that this pandemic is going to cause”, “I am afraid that this crisis will aggravate social inequality in our society”) (1 = “Strongly disagree”, 7 = “Strongly agree”) (*r* = 0.47; *p* < 0.001).

Work–family conflict. Three items from the Netemeyer et al. [29] scale were used (“Indicate your degree of agreement or disagreement with the following statements”: “My work obligations interfere with my family life”, “The time my work demands of me makes it difficult for me to assume my family responsibilities”, “The pressure I have at work makes it difficult for me to assume my family responsibilities”) (1 = “Strongly disagree”, 7 = “Strongly agree”) (α = 0.91).

### 4.5. Organizational Variables

Hours worked. This aspect was determined using a question (“How many hours do you work under these new conditions?”) with three answer options: 1 = “Less than usual”, 2 = “The same”, 3 = “More than usual”; three response options: 1 = “Less than usual”, 2 = “The same”, 3 = “More than usual”.

Organizational resources provided by the organizations during teleworking. These were evaluated through five items (“How do you rate the different services that the organization has provided for you to continue working from home?”: “Training”, “Organizational computer”, “Technical advice given”, “Corporate telephone”, “Access licenses to platforms for video-conferencing or collaborative environments”) (1 = “Nonexistent”, 7 = “Totally adequate”) (α = 0.78). 

Security measures for face-to-face work. These were measured using a seven-item ad hoc scale (“How would you rate the various measures your organization has taken to ensure safety during the pandemic? “Provision of face masks by the company/organization”, “Minimum distance required between workers”, “Gloves provided by the company”, “Provision of means for handwashing”, “Establishment of shifts to avoid crowding of workers”, “Clear information on occupational health measures”, “Structural measures for disinfection of the workspace”) (1 = “Totally insufficient”, 7 = “Totally adequate”) (α = 0.75).

### 4.6. Individual Variables

Ruminative responses linked to the health emergency. Two items adapted from Nolen-Hoeksema and Morrow [54] were used (“Indicate how often you are feeling each of these sensations during confinement”: “Without wanting to, I am invaded by thoughts and images about what we are experiencing with this pandemic”, “I get distracted or have trouble concentrating because of my thoughts about this pandemic”) (1 = “Never”, 7 = “Constantly”) (*r* = 0.62; *p* < 0.001).

### 4.7. Data analysis

After checking the normality and homoscedasticity of the sample, descriptive analyses were carried out. Next, to identify predictors of stress, correlational analyses and multiple hierarchical regressions were conducted. Given that for the organizational variables, specific questions were developed for each work modality, the analyses were carried out in a segregated manner (for professionals who teleworked and those who worked face-to-face). Sociodemographic variables (gender, taking care of a dependent person, level of responsibility in organizations, organization size, and education level) and extra-organizational variables (economic threat, work–family conflict), organizational variables (hours worked, organizational resources provided by the organizations for teleworking and safety measures for face-to-face work), and an individual variable (rumination) were included.

Finally, in order to analyze the modulating role of gender, caregiving, level of responsibility in organizations, and organization size in both telework and face-to-face work, the macro process of Hayes et al. (Dubuque, IA, United States) [55] was used. The Hayes et al. macro process allows for moderation analysis to be conducted in SPSS (IBM, Madrid, Spain). Work stress was introduced into the model as a dependent variable; economic threat, work–family conflict, rumination, and the resources provided by the organizations for teleworking as independent variables; and gender, caregiving, the level of responsibility, and organization size as modulating variables.

## 5. Results

### 5.1. Descriptive According to the Modality of Work

Table 1 presents the variables studied according to work modality. 

### 5.2. Predictors of Work Stress According to Work Modality

Table 2 presents the correlations between work stress and the set of variables studied according to work modality.

Work stress correlated positively and significantly with perceived economic threat, work–family conflict, hours worked, and rumination in both work modalities. Likewise, in both work modalities, stress correlated negatively with the provision of lower levels of organizational resources (for teleworking) and security measures (for working on-site).

The variables that correlated with work stress were then entered into the hierarchical regression analysis, grouping them according to the extra-organizational, organizational, and individual categories in which the variables had been included.

Among teleworkers, work–family conflict, hours spent at work, and rumination were found to be predictors of work stress (see Table 3).

Among face-to-face workers, work–family conflict, security measures, and rumination explained the variance in work stress. In addition, perceived economic threat predicted job stress in a tendential manner (see Table 4).

### 5.3. Moderation Analysis Results

**Figure 1:**
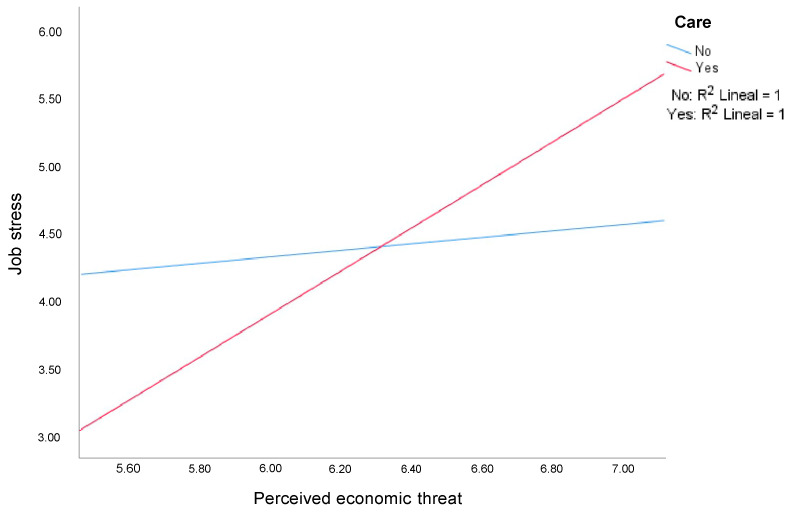
Influence of perceived economic threat on work-related stress as a function of caregiving in a face-to-face work setting.

The caregiving variable modulated the relationship between perceived economic threat and job stress in the face-to-face work modality (β = 1.39, SE = 0.439, t = 3.171, *p* = 0.02). In particular, the positive relationship between perceived economic threat and job stress was more pronounced in those who have dependents under their care (Figure 1).

The model including caregiving and perceived economic threat explained 16% in the variance of job stress (R2 = 0.16, F(3100) = 6.45, *p* = 0.005, f2 = 0.19), and the interaction explained an additional 8% in the variance in job stress (change in R2 = 0.08, F(1100) = 10.13, *p* = 0.01).

**Figure 2:**
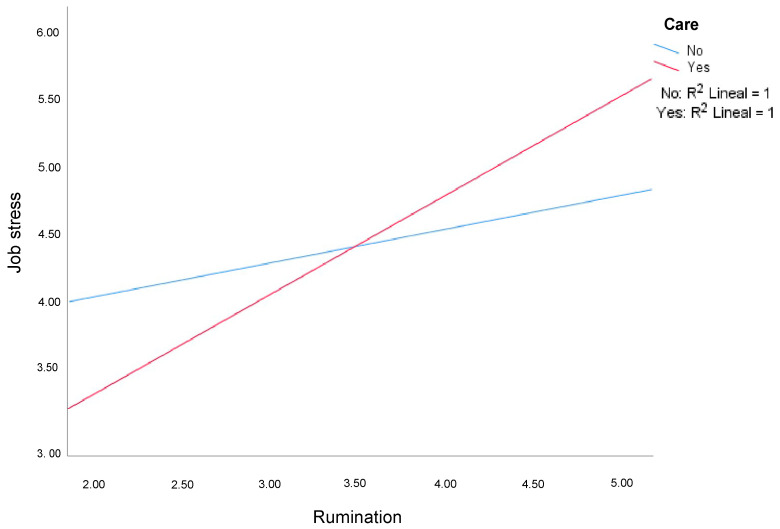
Influence of rumination on work-related stress as a function of caregiving in a face-to-face work setting.

The caregiving variable also modulated the relationship between rumination and job stress in the face-to-face work modality (β = 0.53, SE = 0.23, t = 2.291, *p* = 0.02). The positive relationship between rumination and work stress was also more pronounced in those who have dependents under their care (Figure 2).

The model including caregiving and rumination explained 18% in the variance of job stress (R2 = 0.18, F(3100) = 7.37, *p* = 0.002, f2 = 0.22), and the interaction explained an additional 4% in the variance in job stress (change in R2 = 0.04, F(1100) = 5.13, *p* = 0.03).

In the relationship between work–family conflict and stress, caregiving and gender did not moderate this relationship in any population analyzed. In addition, the level of responsibility in organizations did not moderate the relationship between perceived economic threat and work-related stress. Finally, none of the moderation analyses yielded significant results in the case of the teleworking population.

Finally, in Table 5 are presented a summary of the results of the tested hypotheses. 

## 6. Discussion

In addition to being a health pandemic, COVID-19 has entailed a considerable shift in the working conditions of millions of people globally, modifying habits, ways of relating to work, further altering family reconciliation, and revealing new challenges that both people and organizations have had to face. Given the unprecedented situation created by the health crisis, this study has sought to extend knowledge of the socio-labor conditions faced by those who continued to work, identifying the predictors of stress, taking into account the need to analyze these from a holistic perspective.

Concerning the workers’ stress levels, the mean stress scores exceed the theoretical mean in both populations and in accordance with previous studies [17], and no significant differences were found regarding work modality. Even so, the participants’ stress level is lower than reported in the recently mentioned study [17], which could be due to the time elapsed since the beginning of the confinement and the adaptive capacity developed by the participants over these weeks. Nonetheless, the variables studied seem to explain the stress suffered by workers during confinement. In particular, we found similarities and differences in the variables that could explain the emergence of stress according to work modality and other related modulating variables.

Regarding extra-organizational variables and economic threat, participants showed a high perception of economic threat regardless of working modality. Although, its predictive power for stress was lower than that found in other studies conducted during confinement [24,25]. A possible explanation for this result is the bias of the non-probabilistic sample of this study. That is, people who participated in this study occupied a medium-high hierarchical position in their organizations. In particular, whereas perceived economic threat tendentially predicted stress for those who worked face-to-face, this was not significant for those who teleworked (confirming partially the hypothesis 1). In addition, confirming partially the hypothesis 2-a, it was found that among those who worked face-to-face, those with caregiving responsibilities experienced greater economic threat. At the same time, there was no moderating relationship derived from caregiving responsibilities among those who continued to telework. Thus, being in charge of a dependent person entailed greater economic concern [26], although in this study, this was only confirmed for those who worked in person. We conclude that concerning the sample of this study, employees who are teleworkers with caregiving responsibilities have felt more protected compared to those who worked in person with dependents. Thus, face-to-face workers with dependents felt that they were more vulnerable to the economic situation that could arise from the confinement. These results confirm hypothesis 3, given that perceived economic threat was more predictive of stress in people who worked face-to-face compared with those who teleworked. In accord with previous studies [4,5], these results can be explained by the fact that in our sample, people who teleworked—compared with those who worked in person—have higher hierarchical positions in work organizations. However, there is a need to delve deeper into the reasons for this finding. In fact, contrary to our expectations, the level of responsibility within organizations did not moderate the relationship between stress and perceived economic threat in any work modality (rejecting hypothesis 2-c), and, in the face-to-face modality, workers did not perceive higher levels of economic threat. Further, since we did not obtain answers about the income level of the participants, it was not possible to analyze the role of income level as a modulator of the relationship between economic threats and stress (hypothesis 2-d). 

Additionally, and rejecting hypothesis 2-b, gender did not significantly moderate the relationship between perceived economic threat and stress. Concerning this, although outside of crisis in western countries, gender differences are negligible regarding both perceived and actual unemployment risk. The lower status of women in the labor market, and the sectors in which they predominantly work (tourism, commerce), suggest that women will be more vulnerable to the economic consequences of the pandemic [27]. Thus, to capture the impact of gender, it seems important to consider how this relates to other variables. Therefore, in our study, we think that the lack of gender impact on perceived economic threat could be explained by the fact that men and women hold a similar level of responsibility in the different work modalities.

Concerning the work–family conflict variable, low levels of work–family conflict were found, which could be explained by the fact that our study sample contained a low percentage of participants with caregiving responsibilities (20.6%). Despite the generally low levels of conflict, this was found to predict stress in both work modalities, confirming its predictive role [30] (hypothesis 4). Moreover, in this dimension, no significant differences were found between face-to-face modality and teleworking modality workers. It is likely that confinement would blur the possible advantages of telework in facilitating work–life balance. Even though in the pre-pandemic context, teleworking allowed a better family reconciliation, in confinement context, teleworking was developed in parallel with the closing of schools, which could question its protective role.

Besides, given the challenges of caregiving during confinement, and the fact that such responsibilities fall mainly on women [31,32,56], it should be noted that this relationship was independent of gender and caregiving responsibilities (rejecting hypothesis 5-a and 5-b). Thus, although studies have shown that caregiver people and women [31,32,56] have experienced more difficulties in reconciling work and family life, during confinement, the relationship between productive and reproductive work has also predicted stress in men and in those without dependents.

Regarding organizations, resources provided by the organization to ensure safety in the face-to-face modality were evaluated as moderate, and these were predictors of stress only among those who worked face-to-face (confirming hypothesis 6). This latter finding is consistent with those studies conducted during the pandemic showing that the lower presence of psychological symptomatology is related to confidence and perceived safety in the workplace [49]. This result reinforces the importance of the resources provided by organizations to protect the occupational health of their employees [57]. Besides, and in accord with the literature [11], we found a poor evaluation of the resources allocated to teleworking. Even so, such organizational resources did not influence the level of stress found among those who teleworked (rejecting partially hypothesis 7). Organization size also did not moderate the relationship between organizational resources and stress (rejecting hypothesis 8). Although it is considered necessary for organizations to provide basic resources to adequately carry out telework [2], the results of this study suggest that workers could have been provided with the resources needed to perform their work duties. Moreover, hours worked were only explanatory for stress among those who teleworked (confirming partially hypothesis 7). Additional working hours were primarily reported by those who teleworked. This might be able to explain why this factor predicted stress in telework, but not in face-to-face work. In particular, one-third of the people who teleworked, and one-fifth of those who worked face-to-face, signaled a situation of workload. Thus, it is likely that teleworkers did not work under the appropriate conditions, and that this sudden adaptation to teleworking implied a greater workload for them [16,17,18,19,20,21,22,23,24,25,26,27,28,29,30,31,32,33,34,35,36,37,38,39,40,41,42,43,44,45,46].

Regarding the predictive effect of the individual variable of rumination, despite the ruminative thoughts associated with the pandemic not being high, the results show that this predicted stress in both work modalities (confirming hypothesis 9). In addition, confirming partially the hypothesis 10-b, it was found that among people who worked face-to-face, being in charge of a dependent person led to higher levels of rumination than workers without caregiving responsibilities. In contrast, in the teleworking population, this moderation relationship related to caregiving responsibilities was not identified. This result could be explained by the greater exposure to the disease, and the higher risk of contagion, among face-to-face workers [16], and showed that taking care of a dependent person brought greater fear of contagion among face-to-face workers. The results suggest that working in the face-to-face mode during confinement, and having dependents, led to an especially difficult situation, highlighting the related risks of working face-to-face, as well as the advantages of teleworking over face-to-face working. In this context, it could be understood that those who worked face-to-face had a higher score in rumination. Finally, the study by Molero et al. [51] found that women showed a greater fear of falling ill because they were more vulnerable to COVID-19. However, in our study, regarding ruminative thoughts, no gender influence was detected (rejecting hypothesis 10-a). 

### 6.1. Practical Applications for Organizations 

Identifying the main predictors of stress gives us the opportunity to learn, at the organizational level, about how to deal with these situations, and improve the conditions of the people who work in them. 

Concerning the economic threat and instability regarding the future of work, the concern detected among employees and its tendential explanatory role in the emergence of stress among those who worked face-to-face (particularly among those with care responsibilities) suggests that it is important for organizations to evaluate the best strategies to provide workers with the greatest possible job security in situations such as the one recently experienced. In addition to state assistance, it would be advisable to offer employees transparent information on the organization’s financial situation, and establish participatory mechanisms with the teams where workers are involved in the measures to be established.

With respect to reconciliation, despite the fact that we did not detect a high level of work–family conflict, work–family conflict is a predictive factor for stress in both work modalities. In this sense, and given the risk of new confinements, organizations could establish measures to prevent the burden of reconciliation (of workers with and without dependent responsibilities) from falling exclusively on workers, readjusting the workload to the new scenario, or adapting working hours to ensure the most comfortable conditions for employees. 

Moreover, considering that the number of hours worked predicted stress among the teleworkers, and that we detected an increase in their workload, particularly in the teleworking population, we consider that measures should continue to be implemented to regularize the working conditions of teleworkers. Such measures should be put into place as soon as possible to avoid a situation of stress in employees, and enable digital disconnection. In this regard, progress has already been made in the Spanish State at the legislative level by Royal Decree Law [58]. This law seeks to regulate the relationship between the employee and the organization, obliging them to enter into an agreement, and to record schedules, among other measures. However, as with the case of face-to-face work, we consider that work inspections should play an active role, allowing public institutions to monitor the work that takes place at home.

Finally, we recommend that face-to-face workers should be provided with all the necessary resources and safety assurances. Thus, in possible health crises in the future, organizations should provide all the necessary means to cushion the threat of contagion, and avoid the ruminative thoughts linked to an increase in employee stress. In this regard, it could be helpful to provide workers with a psychologist service. In addition, the results suggest that it would be advisable to pay special attention to people with caregiving responsibilities.

### 6.2. Limitations and Future Lines of Research

Conditioned by the lockdown situation, a non-probabilistic snowballing sampling strategy was used for the collection of the survey. Consequently, the main limitation of this study is that the results could not generalized in the wider population. In this sense, the contributions of this study must be considered in a situated manner.

Another limitation of our research is its cross-sectional design. Since this study was developed to analyze which were the predictors of labor stress during confinement, no pre-lockdown data were collected. Consequently, given the non-longitudinal nature of the study, it is not possible to compare the results with the normal situation. Thus, it has not been possible to conclude whether stress levels and their predictors are due specifically to the confinement situation.

Besides, we considered that analyzing the predictor of stress according to work modalities (face-to-face or teleworking) has given relevant information to delve into in the socio-labor conditions worked during confinement. However, an important limitation of this study was the lack of more precise information about occupations (e.g., the leader of the occupation, the option to choose to work face-to-face or by remote work, or whether they had part-time or full-time jobs), which would have helped us to better interpret the results. Moreover, it would have been interesting to collect more information about the organizations in which people worked, such as organizational culture or climate. Future studies should also analyze the influence of the experience concerning teleworking on job stress [48,59].

Finally, it would also be interesting to delve deeper into why, given the gender imbalance in caregiving responsibilities, and the burden this has placed on workers with dependents during confinement, these factors did not appear to be associated with high levels of stress in the participants in this study. In this sense, incorporating qualitative methodology into this research would have allowed for a better understanding of the experience under study, facilitating a more in-depth analysis of the importance of variables such as gender or caregiving on the relationship between work–family conflict and stress.

## 7. Conclusions

COVID-19 brought us to a state of unprecedented confinement, and this situation, among other things, brought about profound changes in work activity. One of the priorities of public and private organizations was that people continue working. In this context, we sought to know the socio-labor conditions in which people continued working, and what implications they have on employee job stress. Given the sudden changes that arose in work activity, and the crisis at the social level, it should be noted that the people participating in this study did not report high levels of stress. A possible explanation for this result is that employees have the ability to adapt to the situation, or maybe that they have been able to count on resources (psychological, material, or family). 

However, to know what aspects would have to be considered in a future pandemic, we considered it relevant to understand what the origins of the stress were. At this level, we have highlighted the importance of working from an integrated perspective (considering extra-organizational, organizational, and individual variables), taking into account work modality (teleworking or face-to-face working). Although similar levels of stress were identified in the two work modalities, similarities and specificities were identified concerning the predictors of work stress. Thus, both the tension provoked by the work activity on family care, and ruminative thoughts related to the pandemic, were sources of stress in all employees. In addition, (not) decisions and (not) measures taken by the organizations influenced employee’s stress levels. In particular, in the case of those who worked face-to-face, measures implemented by organizations to work safely predicted stress, and in those who teleworked, the additional hours worked. Finally, among those who worked face-to-face, the perceived economic threat had an impact on employee stress. Moreover, the positive relationship between perceived economic threat and rumination, and job stress was more pronounced in those who take care of a dependent person. Consequently, we concluded that people who worked face-to-face were more vulnerable to the effects of the pandemic compared to teleworkers.

## Figures and Tables

**Table 1:** Means, and T de Student and chi-squared test results according to work modality.

Variables	Total *n* = 328	Telework *n* = 175	Face-to-Face Work *n* = 153	T de Student/Chi-Squared	*p*	d de Cohen/Phi
	M(SD)/%	M(SD)/%	M(SD)/%			
Stress at work	4.26(1.37)	4.14(1.32)	4.4(1.43)	−1.595	0.112	−0.19
Perceived economic threat	6.1(1.03)	6.02(1.07)	6.19(.97)	−1.325	0.186	−0.16
Work–family conflict	3.52(1.74)	3.62(1.69)	3.39(1.81)	1.103	0.271	0.13
Organizational resources to telework	-	3.82(1.53)	-			
Safety measures for on-site work	-	-	4.53(1.41)			
Rumination	3.25(1.44)	3.07(1.41)	3.49(1.45)	−2.384	0.018	−0.29
Hours worked	2.04 (0.71)	2.07 (0.78)	1.98 (0.64)			
Less	23.6%	25.5%	21.2%	0.733	0.392	−0.50
Same	48.5%	40%	59.1%	10.701	0.001	0.190
More	27.9%	34.5%	19.7%	8.029	0.005	−0.164

**Table 2:** Correlations between work-related stress and extra-organizational, organizational, and individual variables according to work modality.

Variables	Stress at Work	
	Teleworking	Going to the workplace
Gender	0.106	0.178
Caregiving	0.134	0.075
Level of education	0.100	0.121
Level of responsibility	−0.049	0.098
Organization size	0.083	0.083
Economic threat perceived	0.165 *	0.234 *
Work–family conflict	0.498 **	0.509 **
Organizational resources for teleworking	−0.221 **	
Safety measures for on-site work		−0.478 **
Hours worked	0.245 **	0.340 **
Rumination	0.360 **	0.366 **

Note. **. Correlation is significant at the 0.01 level (bilateral). *. Correlation is significant at the 0.05 level (bilateral).

**Table 3:** Hierarchical regression on work-related stress among teleworkers.

	R ^2^	B Standardized	Sig	95% CI (LL, UL)	Collinearity Statistics VIF Tolerance	
Model 1	0.238					
Perceived economic threat		0.119	0.108	(−0.030, 0.295)	0.967	1.034
Work–family conflict		0.463	0.001	(−0.239, 0.459)	0.967	1.034
Model 2	0.267					
Perceived economic threat		0.156	0.037	(0.011, 0.334)	0.936	1.068
Work–family conflict		0.388	0.001	(0.176, 0.408)	0.840	1.190
Organizational resources		−0.128	0.091	(−0.228, 0.017)	0.904	1.106
Hours worked		0.153	0.040	(0.012, 0.496)	0.934	1.071
Model 3	0.318					
Perceived economic threat		0.075	0.320	(−0.081, 0.248)	0.842	1.188
Work–family conflict		0.340	0.001	(0.142, 0.370)	0.811	1.233
Organizational resources		−0.108	0.141	(−0.207, 0.030)	0.898	1.113
Hours worked		0.193	0.009	(0.083, 0.557)	0.909	1.100
Rumination		0.258	0.001	(0.097, 0.371)	0.817	1.223

**Table 4:** Hierarchical regression on work-related stress among people with face-to-face jobs.

	R ^2^	B Standardized	Sig	95% CI (LL, UL)	Collinearity Statistics VIF Tolerance	
Model 1	0.297					
Perceived economic threat		0.179	0.029	(0.028, 0.495)	0.984	1.016
Work–family conflict		0.505	0.001	(0.264, 0.509)	0.984	1.016
Model 2	0.426					
Perceived economic threat		0.182	0.014	(0.054, 0.477)	0.981	1.019
Work–family conflict		0.359	0.001	(0.154, 0.395)	0.829	1.207
Safety measures		0-.353	0.001	(−0.499, −0.202)	0.918	1.090
Hours worked		0.146	0.057	(−0.011, 0.673)	0.898	1.114
Model 3	0.478					
Perceived economic threat		0.125	0.083	(−0.024, 0.391)	0.928	1.078
Work–family conflict		0.331	0.001	(0.138, 0.368)	0.819	1.221
Safety measures		−0.356	0.001	(−0.495, −0.212)	0.918	1.090
Hours worked		0.106	0.152	(−0.090, 0.571)	0.874	1.144
Rumination		0.248	0.001	(0.103, 0.391)	0.887	1.128

**Table 5:** Summary of results of tested hypotheses.

Hypothesis				Confirm				Partially				Reject			
1															
2-a	2-b	2-c	2-d												
3															
4															
5-a		5-b													
6															
7															
8															
9															
10-a		10-b													

Note: The shading represents the fulfillment of the hypothesis (confirmed, partially confirmed, or rejected).

## Data Availability

The data presented in this study are available on request from the corresponding author. The data are not publicly available due to them containing personal information.
